# Individual and occupational risk factors for knee osteoarthritis: results of a case-control study in Germany

**DOI:** 10.1186/ar3015

**Published:** 2010-05-14

**Authors:** André Klussmann, Hansjürgen Gebhardt, Matthias Nübling, Falk Liebers, Emilio Quirós Perea, Wolfgang Cordier, Lars V von Engelhardt, Markus Schubert, Andreas Dávid, Bertil Bouillon, Monika A Rieger

**Affiliations:** 1 Institute of Occupational Health, Safety and Ergonomics (ASER) at the University of Wuppertal, Corneliusstraße 31, 42329 Wuppertal, Germany; 2 Freiburg Research Centre for Occupational and Social Medicine (FFAS), Bertoldstraße 27, 79098 Freiburg, Germany; 3 Federal Institute for Occupational Safety and Health, Noeldnerstraße 40-42, 10317 Berlin, Germany; 4 Centre for Orthopaedics and Rheumatology, Clinic for General Orthopaedics, Sankt Josef Hospital, Bergstraße 6-12, 42105 Wuppertal, Germany; 5 Department of Trauma and Orthopedic Surgery, University of Witten/Herdecke, HELIOS Hospital Wuppertal, Heusnerstraße 40, 42283 Wuppertal, Germany; 6 Department of Trauma and Orthopaedic Surgery, University of Witten/Herdecke, Hospital Cologne Merheim, Ostmerheimerstraße 200, 51109 Cologne, Germany; 7 Department of Occupational Health and Environmental Medicine, Institute of General Practice and Family Medicine, University of Witten/Herdecke, Alfred-Herrhausen-Straße 50, 58448 Witten, Germany; 8 Institute of Occupational and Social Medicine, University Hospital of Tuebingen, Wilhelmstraße 27, 72074 Tuebingen, Germany

## Abstract

**Introduction:**

A number of occupational risk factors are discussed in relation to the development and progress of knee joint diseases (for example, working in a kneeling or squatting posture, lifting and carrying heavy weights). Besides the occupational factors, a number of individual risk factors are important. The distinction between work-related and other factors is crucial in assessing the risk and in deriving preventive measures in occupational health.

**Methods:**

In a case-control study, patients with and without symptomatic knee osteoarthritis (OA) were questioned by means of a standardised questionnaire complemented by a semi-standardised interview. Controls were matched and assigned to the cases by gender and age. Conditional logistic regression was used in analysing data.

**Results:**

In total, 739 cases and 571 controls were included in the study. In women and men, several individual and occupational predictors for knee OA could be described: obesity (odds ratio (OR) up to 17.65 in women and up to 12.56 in men); kneeling/squatting (women, OR 2.52 (>8,934 hours/life); men, 2.16 (574 to 12,244 hours/life), 2.47 (>12,244 hours/life)); genetic predisposition (women, OR 2.17; men, OR 2.37); and sports with a risk of unapparent trauma (women, OR 2.47 (≥1,440 hours/life); men, 2.58 (≥3,232 hours/life)). In women, malalignment of the knee (OR 11.54), pain in the knee already in childhood (OR 2.08), and the daily lifting and carrying of loads (≥1,088 tons/life, OR 2.13) were related to an increased OR; sitting and smoking led to a reduced OR.

**Conclusions:**

The results support a dose-response relationship between kneeling/squatting and symptomatic knee OA in men and, for the first time, in women. The results concerning general and occupational predictors for knee OA reflect the findings from the literature quite well. Yet occupational risks such as jumping or climbing stairs/ladders, as discussed in the literature, did not correlate with symptomatic knee OA in the present study. With regards to occupational health, prevention measures should focus on the reduction of kneeling activities and the lifting and carrying of loads as well as general risk factors, most notably the reduction of obesity. More intervention studies of the effectiveness of tools and working methods for reducing knee straining activities are needed.

## Introduction

### Background

Suffering from musculoskeletal diseases or disorders is the most frequent reason for absence from work in the western world. The inability to work as a consequence of diseases or disorders of the musculoskeletal system and the connective tissue resulted in 103.6 million days of absence (23.7% of all days of absence) in Germany in 2007. This led to a loss in the gross domestic product of €17.3 billion [1]. One of the frequent impairing disorders of the musculoskeletal system is knee osteoarthritis (OA).

The central pathologic features of OA are the loss of hyaline articular cartilage and changes in the subchondral bone. A number of occupational and nonoccupational risk factors are related to the development and progress of knee OA, with the proportion of radiographic knee OA in men due to job activities reaching 15 to 30% [2]. For reviews on risk factors with different focuses, see [3-11]. Most of the existing studies focus on exercise through sports, individual factors, genetic factors, or occupational factors. Studies including comprehensive data and analysis are rare. The distinction between work-related and other factors is crucial in assessing risk and in deriving preventive measures in occupational health.

### Aim of the study

The aim of the research project ArGon - an acronym for *Arbeitsbedingungen *(working conditions) and *Gonarthrose *(knee OA) - was to find the most parsimonious model considering different occupational factors (for example, kneeling and squatting activities, the lifting and carrying of loads, standing, jumping) and other influencing factors (for example, age, gender, constitutional factors, sports) to predict the occurrence of symptomatic knee OA in Germany.

## Materials and methods

### Study design

The present case-control study was based on the populations of two neighbouring regions in Germany. The hospitals involved in the study are university teaching hospitals. The hospitals were chosen to include a balanced and representative town-country relationship. The urban and rural infrastructure includes a wide range of industrial workers, craftspeople, office workers, managers as well as farmers in the countryside. Cases were recruited from the surgical-orthopaedic wards and from appropriate outpatient clinics; controls were recruited from the accident surgery services of three participating hospitals and were matched with the case group according to age and place of residence.

Both groups filled out a standardised questionnaire, and a standardised patient record was filled out by an orthopaedic surgeon (cases only). In addition, participants with jobs involving lifting and carrying of loads were interviewed. Besides the consecutive recruitment in the hospitals, patients who could not be addressed directly during their hospitalisation were contacted retroactively by the hospital physician. All questionnaires were collected and evaluated in the study centre.

### Instruments

#### Standardised questionnaire

The questionnaire was developed on the results of a literature review [12]. Previous literature (in English and German) was analysed, and relevant risk factors and confounding factors were included in the questionnaire. Hence the questionnaire contained questions about sociodemographic factors, relevant diseases, occupational history, and leisure-time activities. Participants were asked to describe every occupation, every sport, and every other leisure-time activity, and they were asked to indicate the respective duration (in years) and also the number of hours per day and per week. In the work analysis, the amount of different body postures (sitting, standing, walking, kneeling/squatting) as well as the prevalence of certain job characteristics (for example, climbing stairs, jumping, lifting/carrying of loads, time pressure) was assessed.

#### Partially standardised telephone interview

The telephone interview contained detailed questions on the frequency and duration of lifting and carrying for every occupational employment. This interview was conducted if daily lifting or carrying of loads was mentioned in the questionnaire by cases or controls in order to obtain more detailed information about the individual's work tasks.

#### Patient record

The patients' history and the physicians' findings were documented in a patient record including information on general health status, as well as the condition of knee cartilage, meniscus, and ligaments (according to the International Cartilage Repair Society standard). This patient record was filled out by the orthopaedic surgeon treating the patient (cases only).

### Recruitment and inclusion criteria of cases and controls

#### General inclusion criteria

The inclusion criteria were as follows: age between 25 and 75 years, place of residence in the defined vicinity of the participating hospitals, and linguistic and cognitive ability to understand and fill out the questionnaire and to provide informed consent.

#### Additional criteria for the case group

The case group's additional criteria were as follows: knee OA confirmed by either radiological diagnostics (≥grade II on the Kellgren and Lawrence scale [13]) or findings from arthroscopy or open surgery (≥grade III on the Outerbridge scale [14]). Further criteria for inclusion were: diagnosis of knee OA for no longer than 10 years; no previous fractures involving knee joints or injuries of the knee (ligament or cartilage injuries); and no inflammatory or reactive knee joint illnesses.

#### Additional criteria for the control group

The control group's additional criteria were as follows: treatment for an accident due to an external cause (that is, not due to circulatory, metabolic, or neurological disorders), an accident that was not work-related, and no already existing physician diagnosis of knee OA.

### Power of the dataset

Before recruitment, the power of the dataset was estimated with 800 cases and an equal number of controls using EpiManager software [15]. The distribution was thereby assumed to be approximately 60% women and 40% men.

The estimated number of participants could not be achieved within the 24-month period, although finally 739 cases (including 438 females) and 571 controls (including 303 females) could be included. Assuming a prevalence of 10% for kneeling/squatting activities in the population, a significantly higher prevalence (odds ratio (OR) >2) would be detected with a power of approximately 80% in men and 88% in women if there were no confounding factors.

### Analysis

In the first step, cumulative calculation of life doses was determined over all practiced activities and occupations (hours/life, tons/life, or frequency/life). Smoking was summarised in package-years (1 package-year = smoking 20 cigarettes/day for 1 year). The retrospective observation period for the cases ended at the time at which the diagnosis of knee OA was first made. The time difference between the time of inclusion in the study and the time of diagnosis of knee OA for the first time was calculated for all cases. In the controls, the median of this period (3 years) was subtracted from the time point of inclusion in the study in order to calculate the comparable exposure period in the controls.

In total, 180 items (183 in women) derived from the literature were generated (occupational factors, 19 items; sports, 91 items; leisure-time activities, 19 items; medical history, 29 items; individual factors, 22 items (25 in women)).

In the next step, all items were checked for correlation with the outcome (symptomatic knee OA) in bivariate analysis separately for men and women using logistic regression. As most sport activities showed a low prevalence, orthopaedic and accident surgeons as well as a sport physician were asked to group the single activities into categories (for example, activities suitable for prevention of knee OA, activities with impact force on the knee joint, activities with risk for unapparent trauma of the tibiofemoral joint). All of these groups were also correlated separately with the outcome. The strongest correlation was between the outcome and the group of sports with risk for unapparent trauma (in hours/life). This group was used for further analysis.

All items correlating with *P *< 0.2 were selected for further analysis. This procedure was based on the references of Hosmer and Lemeshow [16]. Thirty-six items in men and 39 items in women were found to be in significant association with the outcome (men/women: occupational factors, 16 items/10 items; leisure-time activities, 2 items/3 items; medical history, 12 items/17 items; individual factors, 5 items/7 items; and sports with risk for unapparent trauma, 1 item/1 item). These items were taken into the final multivariable model aimed at describing the most parsimonious model for the occurrence of symptomatic knee OA in Germany (separately for men and women).

In the next step, to form the final model, constant items were transformed into categorical variables for better representation. A further reason for the transformation into categorical variables was the fact that the metric parameters only rarely showed a normal distribution. With the categorisation of the cumulative life doses, the zero group (no exposure at all) was defined as a separate category; the remaining values were then divided into two groups (median split) or into three groups (tertile split), depending upon the remaining group size. The body mass index (BMI) was categorised into the groups of normal weight (BMI = 18.5 to <25 kg/m^2^), overweight (BMI ≥25 to <30 kg/m^2^), obesity grade I (BMI ≥30 to <35 kg/m^2^), obesity grade II (BMI ≥35 to <40 kg/m^2^), and obesity grade III (BMI ≥40 kg/m^2^) according to the definitions of the World Health Organization [17]. Among men, the two groups obesity grade II and obesity grade III were merged, since the number of men was very small with regard to obesity grade III. These categorised groups of exposure were compared in each case with the zero-exposure group.

Owing to an unequal distribution of the age between cases and controls, age-stratified evaluations (five age groups) were carried out. The models were computed with conditional logistic regression using SAS 9.2 (SAS Institute Inc, North Carolina, USA). The most parsimonious models (only significant predictors enclosed, *P *≤ 0.05) for men and women were calculated (successive slimming).

### Ethics

The study protocol [12] was approved by the Ethical Committee of the University Witten/Herdecke (approval number 61/2006). The ethical aspects were in full agreement with the Helsinki Declaration as well as the German Federal Data Protection Act.

## Results

### Description of the sample

**Figure 1:**
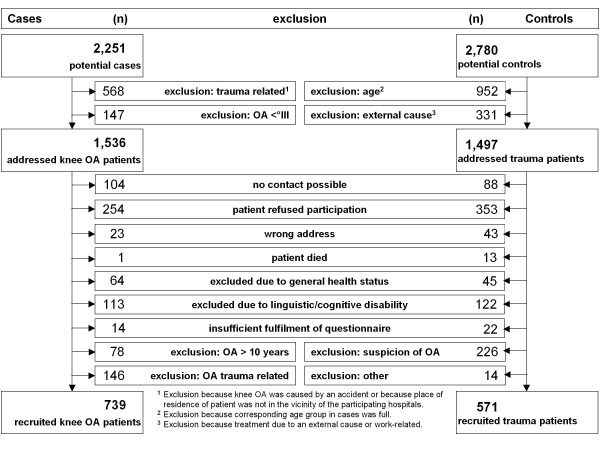
**Recruitment of cases and controls**. OA, osteoarthritis.

In the 24-month recruitment period 2,251 potential cases and 2,780 potential controls were analysed, from which 739 cases and 571 controls could be included in the study (Figure 1). The distribution of the included cases and controls is presented in Table 1.

**Table 1:** Distribution of cases and controls

			Age at inclusion in study (years)		Age used for exposure analysis (years)	
				
	Gender	***n***	Mean	Standard deviation	Mean	Standard deviation
Cases	Female	438	62.0	9.6	59.6	9.8
	Male	301	60.0	11.1	57.1	11.2
Controls	Female	303	57.8	11.8	54.8	11.8
	Male	268	53.9	12.7	50.9	12.7

### Results of exposure assessment

The proportion of exposed and nonexposed subjects among cases and controls with regard to occupational exposures are presented in Table 2.

**Table 2:** Occupational exposure to knee-straining activities: proportion of exposed and nonexposed subjects among cases and controls

			Exposed		Not exposed		No indication	
					
Exposure	Gender	Controls/cases	***n***	%	***n***	%	***n***	%
Sitting	Female	Controls	261	86.2	28	9.2	14	4.6
		Cases	367	83.8	60	13.7	11	2.5
	Male	Controls	228	85.1	34	12.7	6	2.2
		Cases	239	79.4	53	17.6	9	3.0
Standing	Female	Controls	240	79.2	49	16.2	14	4.6
		Cases	363	82.9	64	14.6	11	2.5
	Male	Controls	226	84.4	36	13.4	6	2.2
		Cases	249	82.7	43	14.3	9	3.0
Walking	Female	Controls	249	82.2	40	13.2	14	4.6
		Cases	381	87.0	46	10.5	11	2.5
	Male	Controls	241	90.0	21	7.8	6	2.2
		Cases	266	88.4	26	8.6	9	3.0
Kneeling, squatting	Female	Controls	113	37.3	176	58.1	14	4.6
		Cases	215	49.1	212	48.4	11	2.5
	Male	Controls	118	44.1	144	53.7	6	2.2
		Cases	163	54.5	129	42.5	9	3.0
Climbing stairs	Female	Controls	186	61.4	104	34.3	13	4.3
		Cases	317	72.4	111	25.3	10	2.3
	Male	Controls	181	67.6	81	30.2	6	2.2
		Cases	220	73.1	75	24.9	6	2.0
Jumping	Female	Controls	46	15.3	244	80.5	13	4.2
		Cases	91	20.8	337	76.9	10	2.3
	Male	Controls	79	29.5	183	68.3	6	2.2
		Cases	120	39.8	173	57.5	8	2.7
Lifting/carrying of loads	Female	Controls	101	32.0	144	47.5	62	20.5
		Cases	152	35.7	190	43.4	96	21.9
	Male	Controls	162	60.4	87	32.5	19	7.1
		Cases	196	65.1	83	27.6	22	7.3

The prevalence of sports and leisure-time activities was somewhat equal within cases and controls. Some of the interviewees could not remember the amount and the duration of their activities. Interviewees with and without specifications on the amount and duration of activities are therefore described separately in Table 3.

**Table 3:** Exposure to sports and leisure-time activities

		Controls		Cases		Total	
				
Gender	Exposure	***n***	%	***n***	%	***n***	%
Female	No sports	94	31.0	143	32.6	237	32.0
	Sports - cumulative exposure could be calculated	170	56.1	237	54.1	407	54.9
	Sports - no cumulative exposure could be calculated	39	12.9	58	13.2	97	13.1
Male	No sports	41	15.3	39	13.0	80	14.1
	Sports - cumulative exposure could be calculated	201	75.0	241	80.1	442	77.7
	Sports - no cumulative exposure could be calculated	26	9.7	21	7.0	47	8.3
Female	No leisure time activities	173	57.1	239	54.6	412	55.6
	Leisure time activities - cumulative exposure could be calculated	97	32.0	169	38.6	266	35.9
	Leisure time activities - no cumulative exposure could be calculated	33	10.9	30	6.8	63	8.5
Male	No leisure time activities	154	57.5	154	51.2	308	54.1
	Leisure time activities - cumulative exposure could be calculated	90	33.6	130	43.2	220	38.7
	Leisure time activities - no cumulative exposure could be calculated	24	9.0	17	5.6	41	7.2

Cumulative exposures were calculated for use in logistic regression analysis. For this calculation, only the exposures of the interviewee who could remember the amount and the duration of their activities were taken into account. Missing values were extracted into a separate group (Table 4).

**Table 4:** Categorisation of the cumulative life doses

	Tertile split		
	
	First tertile	Second tertile	Third tertile
Smoking (package-years)			
Female	<9	9 to 20	>20
Male	<16.5	16.5 to 27	>27
Kneeling/squatting (hours/life)			
Female	<3,542	3,542 to 8,934	>8,934
Male	<3,573	3,573 to 12,243	>12,243
Sitting (hours/life)			
Female	<16,031	16,031 to 33,119	>33,119
Male	<15,180	15,180 to 34,960	>34,960
		
	**Median split**		
		
	**Low exposure**	**High exposure**	
		
Lifting and carrying (tons/life)			
Female	<1,088	≥1,088	
Male	<2,214	≥2,214	
Sports with risk for unapparent trauma (hours/life)			
Female	<1,440	≥1,440	
Male	<3,232	≥3,232	

### Predictors of symptomatic knee OA: models

In women, 39 items correlated with the outcome in the bivariate analysis. Based on these outcomes, the most parsimonious model for women was calculated with conditional logistic regression (Table 5). This model contains the variables pain in the knee during childhood, knee OA in close relatives (parents, brother, or sister), malalignment of the tibiofemoral joint, BMI, cumulative kneeling or squatting (in hours/live), smoking (in package-years), cumulative sitting (in hours/life), cumulative daily lifting and carrying (in tons/life), and cumulative sports with risk of unapparent trauma (in hours/life). Beside the occupational exposure, the data for sitting and kneeling or squatting also include housework activities. The reference categories were set to an OR of 1. In the further categories of the variables, the OR is compared with the respective reference category.

**Table 5:** Conditional logistic regression model for women: most parsimonious model

	Item	*n*_tot_	Cases (*n*)	Controls (*n*)	*P *value	Odds ratio	95% confidence interval
Knee pain during childhood	No (R)	623	361	262		1.00	-
	Yes	59	41	18	<0.05	2.08	1.01 to 4.26
Knee OA in relatives	No (R)	408	216	192		1.00	-
	Yes	205	142	63	<0.001	2.17	1.40 to 3.37
Malalignment of the knee	No (R)	624	336	288		1.00	-
	Yes	90	83	7	<0.001	11.54	4.65 to 28.66
Body mass index	18.5 to <25 kg/m^2 ^(R)	255	97	158		1.00	-
	≥25 to <30 kg/m^2^	249	163	86	<0.001	3.21	2.09 to 4.96
	≥30 to <35 kg/m^2^	149	107	42	<0.001	3.55	2.12 to 5.94
	≥35 to <40 kg/m^2^	55	49	6	<0.001	11.58	4.38 to 30.63
	≥40 kg/m^2^	23	20	3	<0.001	17.65	4.50 to 69.23
Smoking	No (R)	391	255	136		1.00	-
	Yes, <9 package-years	117	63	54	NS	0.69	0.40 to 1.17
	Yes, 9 to 20 package-years	114	64	50	NS	1.16	0.67 to 2.03
	Yes, >20 package-years	115	54	61	<0.01	0.43	0.26 to 0.73
Occupation: kneeling or squatting	No (R)	388	212	176		1.00	-
	Yes, <3,542 hours/life	109	62	47	NS	1.50	0.83 to 2.69
	Yes, 3,542 to 8,934 hours/life	110	68	42	NS	1.36	0.78 to 2.37
	Yes, >8,934 hours/life	109	85	24	<0.01	2.52	1.35 to 4.68
Occupation: sitting	No (R)	88	60	28		1.00	-
	Yes, <16,032 hours/life	209	127	82	NS	0.72	0.37 to 1.40
	Yes, 16,032 to 33,119 hours/life	209	122	87	<0.05	0.51	0.26 to 0.99
	Yes, >33,119 hours/life	210	118	92	<0.01	0.39	0.20 to 0.76
Occupation: lifting and carrying	No (R)	263	139	124		1.00	-
	sometimes	65	37	28	NS	0.88	0.44 to 1.77
	Yes, <1,088 tons/life	122	69	53	NS	0.69	0.38 to 1.24
	Yes, ≥1,088 tons/life	121	92	29	<0.01	2.13	1.14 to 3.98
Sports with risk for unapparent trauma	No (R)	570	342	228		1.00	-
	Yes, <1,440 hours/life	81	41	40	NS	0.92	0.48 to 1.75
	Yes, ≥1,440 hours/life	78	50	28	<0.01	2.47	1.31 to 4.65

NS, not significant; (R), reference category.

The highest OR was calculated with rising BMI. Compared with those female participants with normal weight, women with obesity grade I had a higher risk of suffering from symptomatic knee OA (OR, 3.5; 95% confidence interval (CI), 2.1 to 5.9), as did the group of women with obesity grade II (OR, 11.6; 95% CI, 4.4 to 30.6) and women with obesity grade III (OR, 17.6; 95% CI, 4.5 to 69.2) in particular. The presence of a malalignment of the tibiofemoral joint was also associated with symptomatic knee OA (OR, 11.5; 95% CI, 4.7 to 28.7) in women. Within the physical loads, cumulative kneeling and squatting >8,934 hours over life increased the risk of symptomatic knee OA (OR, 2.5; 95% CI, 1.4 to 4.7). Cumulative daily lifting and carrying ≥1,088 tons over life resulted in an OR of 2.1 (95% CI, 1.1 to 4.0). Further risk factors for the development of symptomatic knee OA are genetic predisposition (knee OA in parents, brother or sister: OR, 2.2; 95% CI, 1.4 to 3.4), pain in the knee as a child (OR, 2.1; 95% CI, 1.0 to 4.3), and the practice of injury-prone types of sport with an extent of ≥1,440 hours over life (OR, 2.5; 95% CI, 1.3 to 4.6). A decreasing effect was calculated for smoking (>20 package-years: OR, 0.4; 95% CI, 0.3 to 0.7) and cumulative sitting (OR, 0.5; 95% CI, 0.3 to 1.0) for 16,032 to 33,119 hours over life, and for >33,119 hours over life (OR, 0.4; 95% CI, 0.2 to 0.8).

In men, 36 items correlated with the outcome in bivariate analysis. Based on these outcomes, the most parsimonious model for men was calculated with conditional logistic regression (Table 6). This model contains the variables knee OA in close relatives (parents, brother, or sister), BMI, cumulative kneeling or squatting (in hours/life), and cumulative sports with risk for unapparent trauma (hours/life).

**Table 6:** Conditional logistic regression model for men: most parsimonious model

	Item	*n*_tot_	Cases (*n*)	Controls (*n*)	*P *value	Odds ratio	95% confidence interval
Knee OA in relatives	No (R)	367	170	197		1.00	-
	Yes	109	76	33	<0.01	2.37	1.41 to 3.98
Body mass index	18.5 to <25 kg/m^2 ^(R)	157	48	109		1.00	-
	≥25 to <30 kg/m^2^	240	133	107	<0.001	2.26	1.43 to 3.57
	≥30 to <35 kg/m^2^	128	84	44	<0.001	4.00	2.30 to 6.94
	≥35 kg/m^2^	40	35	5	<0.001	12.56	4.40 to 36.86
Occupation: kneeling or squatting	No (R)	272	128	144		1.00	-
	Yes, <3,574 hours/life	94	48	46	NS	1.70	0.96 to 3.00
	Yes, 3,574 to 12,244 hours/life	94	55	39	<0.01	2.16	1.24 to 3.77
	Yes, >12,244 hours/life	94	61	33	<0.01	2.47	1.41 to 4.32
Sports with risk for unapparent trauma	No (R)	218	109	109		1.00	-
	Yes, <3,232 hours/life	168	82	86	NS	1.57	0.98 to 2.52
	Yes, ≥3,232 hours/life	168	104	64	<0.01	2.58	1.59 to 4.17

NS, not significant; (R), reference category.

Similar to the women, the highest OR appeared with rising BMI in men. Compared with those male participants with normal weight, men with obesity grade I had a higher risk of suffering from symptomatic knee OA (OR, 4.0; 95% CI, 2.3 to 6.9), as did men with obesity grade II or obesity grade III (BMI ≥35 kg/m^2^: OR, 12.6; 95% CI, 4.4 to 35.9). Within the physical loads, cumulative kneeling and squatting for 3,574 to 12,244 hours over life led to an increased risk to suffer from symptomatic knee OA (OR, 2.2; 95% CI, 1.2 to 3.8). The risk increased even further when cumulative kneeling or carrying was >12,244 hours (OR, 2.5; 95% CI, 1.4 to 4.3). Lifting and carrying as well as pulling and pushing of loads did not result as a predictor for symptomatic knee OA in men. Further factors of risk were the genetic predisposition (knee OA with parents, brother, or sister: OR, 2.4; 95% CI, 1.4 to 4.0) and the practice of injury-prone sports ≥3,232 hours (OR, 2.5; 95% CI, 1.6 to 4.2).

## Discussion

### Symptomatic knee OA and occupational factors

#### Symptomatic knee OA and kneeling/squatting

In the present study, an OR of 2.5 (95% CI, 1.4 to 4.7) for accumulated kneeling and squatting >8,934 hours over life in women was calculated. In men, the OR for kneeling/squatting for 3,474 to 12,244 hours over life was 2.2 (95% CI, 1.2 to 3.8), and the OR for kneeling/squatting for >12,244 hours over life was 2.5 (95% CI, 1.4 to 4.3). These results indicate an effect of kneeling/squatting on the occurrence of symptomatic knee OA in both genders.

In 2005 Jensen calculated an individual exposure from the amount of knee-straining activities and the number of years in the trade within a collective of floor layers, carpenters and compositors. The ORs for knee complaints and radiographically determined knee OA were 3.0 (95% CI, 0.5 to 17.2) in the low-exposure group, 4.2 (95% CI, 0.6 to 27.6) in the medium-exposure group, and 4.9 (95% CI, 1.1 to 21.9) in the high-exposure group compared with the zero-exposure group [18]. D'Souza and colleagues reported on an analysis of the US national survey (Third National Health and Nutrition Examination Survey (NHANES III)) and used ergonomists' ratings of job categories to describe relationships between work activities and symptomatic knee OA [19]. A significant exposure-response relationship was found between symptomatic knee OA and kneeling in men but not in women. Within a German case-control study, the OR of having radiographically confirmed knee OA was 2.4 (95% CI, 1.1 to 5.0) within the group with cumulative exposure to kneeling and squatting >10,800 hours compared with unexposed subjects [20].

To our knowledge, only one study investigating the dose-response relationship of cumulative kneeling or squatting and knee OA found no correlation [21]. In this study, however, the daily exposures of kneeling and squatting were asked dichotomously (>1 hour/day or ≤1 hour/day) and then multiplied by exposure years, so these results might be imprecise.

In sum, our results support the presumptions that there is a dose-response relationship between knee-straining work activities and symptomatic knee OA, and that this relationship exists also in women.

#### Symptomatic knee OA and lifting and carrying of loads

In the present study, an OR of 2.1 (95% CI, 1.1 to 4.0) could be derived in women for lifting and carrying of least 1,088 tons over life. This correlation was not significant in men.

In the study by D'Souza and colleagues mentioned above, a significant trend in heavy lifting and severe symptomatic knee OA was detected in both genders [19]. Coggon and colleagues calculated an OR of 1.7 (95% CI, 1.2 to 2.6) for regular lifting and carrying of loads >25 kg (men and women considered in common) [21]. In the study by Seidler and colleagues, lifting and carrying of loads was significantly associated with knee osteoarthritis [20]. The dose-response relationship between lifting and carrying of loads and knee OA was described with an OR of 2.0 (95% CI, 1.1 to 3.6) in the exposure group of 630 to <5,120 kg-hours over life, up to an OR of 2.6 (95% CI, 1.1 to 6.1) in the highest exposure group (>37,000 kg-hours over life) in men. Jensen also investigated the correlation between knee OA and lifting and carrying of loads in her review [8]. She concluded that there is moderate evidence of a dose-response relationship between the lifting and carrying of loads and knee OA.

Our results support the current position that there is moderate evidence of a dose-response relationship between the lifting and carrying of loads and symptomatic knee OA.

#### Symptomatic knee OA and jumping down or climbing stairs or ladders

In the present study, neither in men nor in women could a correlation between jumping or climbing stairs and symptomatic knee OA be described. McAlindon and colleagues examined a subset of the Framingham Heart Study cohort [22]. They also did not detect effects of climbing stairs. Mounach and colleagues reported in their case-control study that climbing stairs >50 steps/day was associated with a decreased risk of knee OA (OR, 0.5; 95% CI, 0.3 to 0.9) [23]. In contrast, Cooper and colleagues reported an increased OR in people climbing >10 flights of stairs per day (OR 2.7, 95% CI, 1.2 to 6.1) [24]. Sandmark and colleagues described an increased OR (OR, 2.7; 95% CI, 1.7 to 4.1) for jumping in men, but not in women [25]. In the same study, a slightly increased but predominantly nonsignificant OR was described for climbing stairs in both genders. Manninen and colleagues referred to their results of a case-control study wherein climbing already at a medium level of exposure was associated with an increased risk of knee OA among men (OR 3.1; 95% CI, 1.3 to 7.5) [26]. Although in laboratory analyses Sahlström and colleagues identified that jumping down or climbing stairs and ladders revealed a significant increase in movement in the knee compared with normal walking [27], the effect of these exposures on the knee cartilage remains unclear. Our results could not support either of these effects.

#### Symptomatic knee OA and other work factors

In the present study, a correlation between symptomatic knee OA and further work factors (piece-work, time pressure, hand-arm or whole-body vibration, manual handling of heavy tools, working in wetness, coldness, or heat) could not be found. Elsner and colleagues described significant associations between knee OA and some of the work factors just mentioned [28]. In men, hand-arm vibration (OR, 2.8; 95% CI, 1.2 to 6.4) as well as working under wet/cold conditions and/or draught (OR, 2.0; 95% CI, 1.2 to 3.8) were associated with knee OA, but not in women. In women, manual handling of heavy tools (OR, 6.1; 95% CI, 2.0 to 20.1) was associated with knee OA, but not in men. Sandmark and colleagues described a slightly increased but nonsignificant OR for vibration in men, but no effect in women [25]. To conclude, there seems to be low evidence for the effect of additional working factors on the knee, but few studies dealing with these topics are available. Our results do not support the results of Sandmark and colleagues [25] and of Elsner and colleagues [28].

### Symptomatic knee OA and individual factors

#### Symptomatic knee OA and body mass index

Of all the factors observed in the present study, the increase of the BMI correlated strongest in both genders. As stated above, compared with those with normal body weight, an OR up to 12.6 (95% CI, 4.4 to 35.9) in men with obesity grade II or obesity grade III and up to 17.6 (95% CI, 4.5 to 69.2) in women with obesity grade III was calculated. These findings are in compliance with common literature that describes obesity as a major risk factor in the occurrence of symptomatic knee OA.

Anderson and Felson calculated an OR for overweight (OR, 1.7; 95% CI, 1.1 to 2.8), for obesity grade I (OR, 4.8; 95% CI, 2.8 to 8.3), and for obesity grade II + III (OR, 4.5; 95% CI, 1.8 to 11.2) compared with normal body weight in men [29]. In women, the OR was also calculated for overweight (OR, 1.9; 95% CI, 1.2 to 2.9), for obesity grade I (OR, 3.9; 95% CI, 2.6 to 5.7), and for obesity grade II and obesity grade III (OR, 7.4; 95% CI, 5.2 to 10.5), compared with woman with normal weight.

A recent longitudinal study shows that, compared with subjects with a normal BMI, those who were obese (BMI 30 to <35 kg/m^2^) or very obese (BMI ≥35 kg/m^2^) were at an increased risk of incident knee OA (relative risk, 2.4 and 3.2, respectively; *P *for trend <0.001) [30]. Among others, the relevance of BMI was confirmed by Cooper and colleagues (OR, 3.3; 95% CI, 1.6 to 6.9 for BMI ≥25 kg/m^2 ^compared with those with BMI <25 kg/m^2 ^among both genders) [31], by Dawson and colleagues (OR, 36.4; 95% CI, 3.1 to 432.0 for BMI ≥25 kg/m^2 ^compared with those with BMI <25 kg/m^2 ^among both genders) [32], and by Liu and colleagues (OR, 10.5; 95% CI, 7.9 to 14.1 for BMI ≥25 kg/m^2 ^compared with those with BMI <25 kg/m^2 ^among both genders) [33].

Hartmann and Seidel examined data from male construction workers [34]. They calculated the OR for overweight (OR, 1.2; 95% CI, 1.1 to 1.3), for obesity grade I (OR, 1.5; 95% CI, 1.3 to 1.7), for obesity grade II (OR, 1.6; 95% CI, 1.2 to 2.1), and for obesity grade III (OR, 1.8; 95% CI, 1.0 to 3.0) compared with men of normal weight. Liu and colleagues further reported that about 69% of the knee joint replacements in their study sample were to be assigned to overweight causes [33].

According to the results of Wang and colleagues [35], the risk of primary knee and hip joint replacement due to OA relates to both adipose mass and central adiposity. This relationship suggests that both biomechanical and metabolic mechanisms associated with obesity contribute to the risk of joint replacement, with stronger evidence at the knee rather than at the hip.

Our results support these existing results. We could clearly find a strong correlation between increasing BMI and symptomatic knee OA.

#### Symptomatic knee OA and malalignment of the tibiofemoral joint

In the data from the present study, the existence of malalignment of the tibiofemoral joint was associated with symptomatic knee OA in women only (OR, 11.5, 95% CI, 4.7 to 28.7 compared with women without malalignment of the knee).

Malalignment of the knee has rarely found consideration in the relevant epidemiologic literature [11]. Schouten and colleagues published their results of a 12-year follow-up study in 1992 [36]. Besides other factors, previous malalignment of the tibiofemoral joint (OR, 5.1; 95% CI, 1.1 to 23.1 compared with people without malalignments) was determined as a prognostic factor for development of knee OA. Greinemann wrote in his 1983 study among mine foremen that slight malalignment of the tibiofemoral joint did not promote knee OA [37]. A high position of the patella, however, might be an aggressive prearthritic deformity according to the results of that study. Unfortunately, the position of the patella was not assessed in the present study.

Our results support the findings of the current review by Tanamas and colleagues [11], in which malalignment of the tibiofemoral joint was found to be an independent risk factor for the progression of symptomatic knee OA.

#### Symptomatic knee OA and genetic predisposition

In both genders, knee OA within parents, brothers, or sisters was a significant predictor for symptomatic knee OA in the investigated person. The OR was 2.2 (95% CI, 1.4 to 3.4) in women and 2.4 (95% CI, 1.4 to 4.0) in men.

Cooper and colleagues described an OR for the heredity of knee OA of 2.7 (95% CI, 1.3 to 5.5) for both genders combined [24]. The influence of genetic factors for the development of knee OA was the focus of the work group of Spector and colleagues in several studies. In 1996 they published a study among female twins in which they prove a genetic effect for knee and hand OA [38]. The intraclass correlation of a radiographic OA score in identical pairs was 0.64 compared with nonidentical pairs (0.38). In 2004 Spector and MacGregor summarised their findings about the influence of genetic factors of OA derived from classic twin studies [6]. They indicated that the influence of genetic factors was 39 to 65% in knee/hand OA, 60% in hip OA, and 70% in spine OA. According to the authors, therefore, about one-half of OA can be explained by genetic factors [6].

In our study, the assessment of genetic influences was conducted only by the question about knee OA within parents, brothers, or sisters. As described above, the OR clearly increased for both men and women in connection to genetic predisposition. Our results are in compliance with the results of the authors mentioned above.

#### Symptomatic knee OA and smoking

The factor of smoking (here measured in package-years) was negatively associated with symptomatic knee OA in women (smoking >20 package-years). This phenomenon has been discussed several times in other studies.

Felson and colleagues described this phenomenon in an overview article [39] after they detected the negative association between smoking and knee OA when evaluating two large datasets derived from the First National Health and Nutrition Examination Survey (NHANES I) [29] and from the Framingham Heart Study [2]. It appears that smoking or some unidentified factor correlated with smoking modestly protects against the development of knee OA. As a possible explanation, Gullahorn and colleagues reported that, according to their study results, nicotine upregulates glycosaminoglycan and collagen synthetic activity of articular chondrocytes [40]. The findings about the correlation between smoking and knee OA were summarised by Elloumi and Kallel [41]. They concluded that smoking would have a modest protective effect against the development of OA. This protective effect would be widely supported by the anabolic activity that nicotine carries on the chondrocytes of the articular cartilage. Given the dangers associated with nicotine and smoking, however, one cannot recommend tobacco as a prevention factor for OA [41]. Our results are in compliance with the results of these authors.

#### Symptomatic knee OA and sports

In our study when looking at sports with a risk of unapparent knee trauma, cumulative sports (addition of hours over life of all these kinds of sports) was observed to be a relevant factor for symptomatic knee OA. The OR was 2.5 (95% CI, 1.3 to 4.6) in women performing ≥1,440 hours over life and was 2.6 (95% CI, 1.6 to 4.2) within men having performed ≥3,232 hours over life, compared with persons without any sporting activities.

Participation in physical activity is widely accepted to be associated with physical, psychological, and social benefits [42]. In the literature, few studies could be identified that investigated the correlation between sports history and the development of knee OA. In a review by Gross and Marti, the evidence of the correlation between OA and sports was described as moderate [5]. They concluded that very intensive sports exercise can lead to a low-grade increase in the risk for hip and knee OA (ball and strength sports in particular). In very active runners, the risk of OA also increases. The risk of OA at the weight-bearing joints (hip and knee) might be increased by extremely intensive and long-time sports activity, but might not be predominant in the majority of the population engaging in sports, since the amount of sports activity is lower. A recent review [9] detected that some studies had reported an association between physical activity and a risk for knee OA [43-45], and that other studies had shown physical activity may have no effect [46,47] or may even protect the knee joint from degenerative changes [48,49].

In contrast to our results, Manninen and colleagues referred to a comparative analysis of different kinds of sports and knee OA in a case-control study [50]. The OR for knee arthroplasty decreased to 0.9 (95% CI, 0.3 to 2.6) in men with a low number of cumulative exercise hours and to 0.4 (0.1 to 0.95) in those with a high number of cumulative exercise hours, with a history of no regular physical exercise as the reference. For the women, the corresponding ORs were 0.6 (0.3 to 0.93) and 0.6 (0.3 to 0.98). The authors concluded that recreational physical exercise was associated with a decrease in the risk of knee OA [50]. Urquhart and colleagues concluded that certain types of exercise had different effects on different people [9]. Rather than a uniform approach to the implementation of physical activity, individually tailored exercise programmes were needed to allow exercise to be carried out safely.

On the basis of our results, we support the necessity for further investigations on the relationship between physical exercise and symptomatic knee OA.

### Strengths and weaknesses of the present study

#### Strengths of the study

The principle strength of the present study lies in the high power of the dataset. Another strength is the extensive anamnesis, incorporating occupational factors as well as individual factors and leisure-time activities. Additionally, compared with other case-control studies in Germany [51], the response rate is quite high (73.2% in cases and 65.4% in controls). Compared with international studies, however, the response rate is moderate and nonresponse bias may have influenced the results.

#### Weaknesses of the study

##### Characteristics of the study sample

Points of concern are the unequal number of and the unequal age distribution of cases and controls. Owing to this unequal distribution, age-stratified evaluations were carried out to minimise this potential bias. Possible effects of occupational tasks indicated rarely may have been missed due to the fact that the initially calculated sample size could not be reached.

##### Exposure assessment by self-report

According to the study design, exposure assessment had to be assessed retrospectively by self-report - recall bias may therefore occur. People affected by pain in the knee may have overestimated the influencing factors (for example, kneeling or having pain already in childhood). We tried to deflect respondents from the topic of knee OA during recruitment and survey. Cases and controls were told to participate in a general study about the musculoskeletal system; however, cases might have overestimated relevant exposures. Besides, data from the literature suggest that straining activities (such as kneeling) seem to be overestimated in retrospective exposure assessment by self-report. The overestimation with regard to kneeling activities reached up to 30 to 45% on comparing observation and self-reporting immediately after the work shift [52,53]. Since the exposure requested in the present study dated back many years, the overestimation might be even higher. The amount of this recall bias could not be determined, however, as objective data (for example, observation or exposure measurements) on the participants' former activities were not available.

##### Selection of cases

The injury of joint structures, such as the menisci or cruciate ligaments, is a known risk factor for the development of knee OA [9,54]. Meniscal injuries are the most common injuries to the knee [55]. In our study, we strictly excluded all cases that reported previous knee trauma. Yet undetected or unremembered knee injuries might have been prevalent in cases and may have biased the results.

##### Suitability of the control group

The selection of controls was discussed with the advisory board of the present study during development of the study design. Generally, a primarily defined study base is preferred in case-control studies. In Germany, research groups often make use of the database of the public registry office of individual cities in order to recruit population-based controls. With this database, a nearly unbiased sample of a defined region can theoretically be obtained. Yet this method of recruitment can also be disadvantageous, since response rates often turn out to be very low [51]. In addition, controls may return only incomplete questionnaires as their motivation for participation may be lower than in the case group. The data from controls may thus not only be unrepresentative for the general population but also less informative than those of the case group.

Cases were selected from hospitals in the study. The use of this secondary study base was necessary as register data regarding patients with knee OA are not available in Germany. Controls were consistently also collected as hospital-based, addressing the accident surgery wards. The setting for recruitment was therefore the same for both cases and controls. This is crucial, as similarity between recruiting cases and controls is the most important factor [56].

The surgery in Germany is free of charge and patients choose their hospitals, so there should be no bias in selection of the hospitals. The patients were personally contacted by their treating physician. We assumed that this approach may have lead to an essentially higher response rate and higher quality of data than in controls from the public registry database.

The degree to which the hospital control sample is representative of the general population was assessed with respect to occupation, general health status (prevalence of myocardial infarction, apoplexy, hypertension, diabetes, cancer and concussion), education, and smoking habits using databases (the Federal Health Survey 1998 (BGS '98) [57], employment data of the regional Federal Employment Office, and a community-based health study - the Dortmunder Gesundheitsstudie [58] - which was run simultaneously in the same geographical region by other research institutes).

As cases and controls were addressed consecutively and in retrospect, the response rate and general health status of the subgroups were compared in order to control for any bias with respect to the recruitment strategy.

In all of these comparisons, no relevant differences were covered - the results should therefore be generalizable to the general population in the region observed.

## Conclusions

Occupational and nonoccupational risk factors play an important role in the aetiology of symptomatic knee OA. Against the background of a wide variety of discussed risk factors for knee OA, the ArGon study provided the possibility to analyse a large amount of these possible different predictors in multivariable conditional analyses for men and women. In women, for the first time, a dose-response relationship between different predictors and the occurrence of symptomatic knee OA could be described. In both men and women, the relevance of occupational factors as well as nonoccupational and constitutional predictors could be shown.

It is likely that, as in other chronic diseases, these risk factors are either synergistic or additive, and each has a graded relationship to OA risk (for example, the more obese, the higher the risk). Those at highest risk have more than one risk factor [59]. Among the risk factors taken into account in the present study, only a few are modifiable. According to the results of our study, prevention measures in the occupational field should focus on the reduction of kneeling activities as well as the reduction of lifting and carrying. Aside from the aspects of working conditions, prevention should focus on the reduction of obesity. According to the results of Niu and colleagues, obesity was a risk factor for the incidence of but not for the progression of knee OA [30]. These results underline the importance of the early prevention and reduction of obesity.

The importance of preventive behavioural approaches such as weight management and workplaces designed to limit joint overuse was also postulated after analysing the data of the First National Health Survey in Germany [60]. Jensen and Friche reported on an interventional study where information, education and training in the use of new tools and working methods for the purpose of reducing knee strain and knee complaints were implemented in floor layers [61]. The evaluation after 2 years showed that 38% used the new working methods weekly or daily, compared with 37% 3 months after the courses and 10% before. Among controls, only 16% had used the new working methods weekly or daily. The risk of knee complaints was more than double among floor layers who had used the new working methods for less than 1 year, compared with those who had used them more. More well-designed intervention studies on the effectiveness of tools and working methods for the purpose of reducing knee-straining activities are needed. In addition, the implementation of knee-strengthening exercises in worksite health-promotion programmes should be evaluated.

## Abbreviations

BMI: body mass index; CI: confidence interval; OA: osteoarthritis; OR: odds ratio.

## Competing interests

The authors declare that they have no competing interests.

## Authors' contributions

AK, HG, BB, and MAR conceived and designed the study, AK and MAR prepared the manuscript. In addition, EQP, WC, LVvE, MS, and AD were involved in the execution of the study and the writing of this manuscript. MN gave assistance in epidemiological issues and performed parts of the statistical analyses. FL represents the funding body, initiated the study, and was closely involved in the planning and development of the study design. All authors read and approved the final manuscript.
